# Surface roughness effects on aluminium-based ultraviolet plasmonic nanolasers

**DOI:** 10.1038/srep39813

**Published:** 2017-01-03

**Authors:** Yi-Cheng Chung, Pi-Ju Cheng, Yu-Hsun Chou, Bo-Tsun Chou, Kuo-Bin Hong, Jheng-Hong Shih, Sheng-Di Lin, Tien-Chang Lu, Tzy-Rong Lin

**Affiliations:** 1 National Taiwan Ocean University, Department of Mechanical and Mechatronic Engineering, Keelung 202, Taiwan; 2 Academia Sinica, Research Center for Applied Sciences, Taipei 115, Taiwan; 3 National Chiao Tung University, Institute of Lighting and Energy Photonics, Tainan 711, Taiwan; 4 National Chiao Tung University, Department of Photonics, Hsinchu 300, Taiwan; 5 National Chiao Tung University, Department of Electronics Engineering, Hsinchu 300, Taiwan; 6 National Taiwan Ocean University, Institute of Optoelectronic Sciences, Keelung 202, Taiwan

## Abstract

We systematically investigate the effects of surface roughness on the characteristics of ultraviolet zinc oxide plasmonic nanolasers fabricated on aluminium films with two different degrees of surface roughness. We demonstrate that the effective dielectric functions of aluminium interfaces with distinct roughness can be analysed from reflectivity measurements. By considering the scattering losses, including Rayleigh scattering, electron scattering, and grain boundary scattering, we adopt the modified Drude-Lorentz model to describe the scattering effect caused by surface roughness and obtain the effective dielectric functions of different Al samples. The sample with higher surface roughness induces more electron scattering and light scattering for SPP modes, leading to a higher threshold gain for the plasmonic nanolaser. By considering the pumping efficiency, our theoretical analysis shows that diminishing the detrimental optical losses caused by the roughness of the metallic interface could effectively lower (~33.1%) the pumping threshold of the plasmonic nanolasers, which is consistent with the experimental results.

Plasmonic devices have the ability to significantly confine light on the nanoscale and improve the interaction between light and matter^1^. In past decades, several applications of plasmonic devices have emerged in various fields, such as optical integrated circuits, sensing, optical storage, and photovoltaic devices^2^,^3^,^4^,^5^. During the past years, plasmonic cavities surrounded by noble metals have been successfully used in nanolasers to provide a way to reach the diffraction limit^6^,^7^,^8^,^9^,^10^. However, large losses from metals seriously limit the practical applications for surface plasmon polaritons (SPPs). The optical characteristics of metallic materials therefore play an important role in achieving plasmonic lasing. For metallic films with high degrees of roughness, the dispersion relations can be remarkably changed around frequencies near the surface plasmon frequency^11^,^12^. Even highly enhanced and localised surface plasmons will be induced. In order to minimise the considerable optical losses from surface plasmon waves on rough interfaces, several loss mechanisms should be considered, including (1) extrinsic radiation and scattering losses and (2) intrinsic ohmic damping and extinction of localised SPPs in the metals^8^. For example, intrinsic ohmic damping and extrinsic scattering loss can crucially compromise the propagation length of surface plasmon waves with wavelengths in the ultraviolet (UV) regime. Consequently, the surface roughness and crystalline quality of the metallic layer is the essential factor in determining the performance of plasmonic nanolasers or cavities^13^. Some plasmonic nanocavities and nanolasers on high quality metallic films have been realised and have shown superior lasing characteristics^6^,^8^. Nevertheless, not many researchers have made theoretical investigations or quantitative evaluations of the plasmonic effect of the surface roughness of metallic interfaces, especially for its influence on plasmonic applications, including nanocavities and nanolasers.

To investigate the effect caused by surface roughness, we use two different methods to fabricate Al thin films with different roughness. The sample fabricated by molecular beam epitaxy system is single-crystalline (sample A) and the sample fabricated by electron-gun evaporator is polycrystalline (sample B). In the proposed structures, the hybrid plasmonic gap modes, concentrated inside the dielectric gaps between the gain material nanowires and metallic interfaces, are the most promising modes for lasing^6^,^7^,^8^,^9^,^10^,^13^. Since the guided plasmonic mode profiles strongly overlap with the thin gaps, the increase in intrinsic modal loss caused by surface scattering and ohmic damping from SPPs on the rough metallic surface can be significant. We therefore theoretically analyse the effective dielectric functions of Al interfaces (*ε*(ω)) by the modified Drude-Lorentz model^14^ and reflectivity measurements. The surface roughness, which was experimentally obtained by means of atomic force microscopy (AFM), was adopted for evaluating the effective permittivity. Our results reveal that the increase in surface roughness corresponds to stronger intrinsic damping and hence a larger value of Im[*ε*_Al_(ω)]. Using both sets (sample A and sample B) of effective permittivity, we discuss the discrepancy of SPP behaviours on the different metallic interfaces. In addition, we analysed the characteristics of guided hybrid plasmonic modes in the proposed plasmonic structures by finite-element methods (FEM). Further, the pumping efficiency, defined as how the input optical pumping power is coupled to the plasmonic cavity modes, can also be affected by the roughness. We accordingly solve for the threshold gains and pumping efficiencies of the cavity modes to estimate the corresponding pumping threshold for two cases. Our results show that sample A interfaces significantly reduce the threshold gain in comparison to sample B interfaces, which agrees well with the experimental measurements. Our study provides a systematic analysis to evaluate the characteristics of plasmonic nanolasers on metallic interfaces with certain degrees of roughness.

## Results and Discussion

### Investigation of Al metals with different degrees of surface roughness

**Figure 1:**
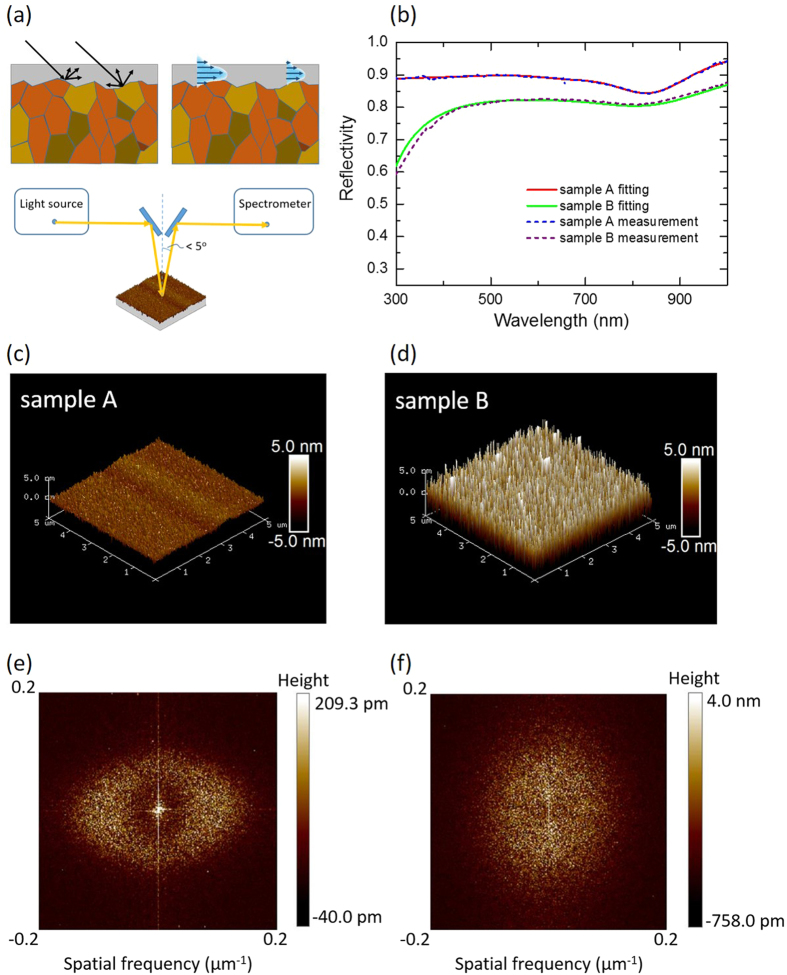
(**a**) The schematic diagram of scattering on the surface and a set of experimental devices for measurement of reflectivity spectra. (**b**) The measured and fitted reflectivity spectra for sample A and sample B films. (**c**,**d**) 5 × 5 μm^2^ atomic force microscope images of the sample A and sample B films. (**e**,**f**) The FFT spectra for sample A and sample B AFM images.

The schematic of optical losses in the plasmonic nanolaser induced by surface roughness is shown in Fig. 1a. Surface roughness on the Al film can result in scattering losses in the plasmonic nanolasers after deposition of the dielectric spacer layer. Scattering losses induced by rough metallic surfaces include Rayleigh scattering, surface roughness scattering, and grain boundary scattering. The surface roughness can induce Rayleigh scattering, which may reduce the portion of photons absorbed by the gain medium, and result in a lower pumping efficiency of the plasmonic nanolasers. On the other hand, when SPPs propagate on the metal-dielectric surface, the rough surface can lead to surface roughness scattering and grain boundary scattering. Both SPP scatterings not only enhance the propagation loss but also suppress formation of the standing wave in the nanolaser cavity. Due to the small skin depth of Al, light interacts mainly with the top layer of Al. As a result, the effects of surface roughness on the dielectric properties of Al films can strongly affect the optical losses of the SPPs. It is essential to control these three kinds of scattering losses to achieve lasing with high performance.

Figure 1a also shows the schematic setup (N&K 1500) for reflectivity measurements on two aluminium samples, sample A and sample B. We measured the reflectivity spectra in the wavelength range from 300 to 1000 nm with a spot size of about 1 mm. These are shown as dashed lines in Fig. 1b. The reflectivity dip around 800 nm corresponds to the absorption of interband transitions in Al. In comparison, sample B has a lower reflectivity than sample A from UV to near-infrared wavelengths, especially in the region below 400 nm. This significant difference is probably due to the surface roughness and the grain boundaries of the Al film, which can induce scattering loss and absorption loss of light.

To understand the effect of Rayleigh scattering for reflectivity measurements induced by surface roughness, we first measured the surface roughness by atomic force microscopy (AFM) as shown in Fig. 1c,d for sample A and sample B, respectively. The surface morphology of sample B is relatively rough; several spikes are present in the image, and the root-mean-square (RMS) roughness of the sample B is 2.5 nm, which is 3.8 times larger than that of the sample A (0.65 nm). By using the degree of surface roughness, we estimate the drop in reflectivity on interfaces due to the Rayleigh scattering^15^ (as shown in the Supporting Information Figure S1). However, only a small drop in reflectivity on the short wavelength side is observed due to the small scale of the surface roughness compared to the incident wavelength, indicating that the discrepancies in reflectivity for our samples on the short wavelength side are not dominated by Rayleigh scattering.

To understand the surface roughness effect of metallic films for SPP modes, we use the modified Drude-Lorentz model considering scattering losses to get the effective dielectric functions of different Al samples. In our previous study^13^, we extracted the permittivity of Al films by fitting the measured reflectivity spectra with the Drude-Lorentz model. However, a discrepancy exists between the model fitting results and measured reflectivity spectra for UV wavelengths. This makes it difficult to understand the optical properties of nanolaser devices with different metal qualities. To investigate the surface roughness effects of our sample A and sample B, we adopt the further modified Drude-Lorentz model by including electron scattering effects in vertical and lateral directions at the Al surface. Since the modified Drude-Lorentz model can be expressed as^14^


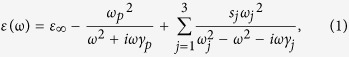


where *ε*_∞_ is the background permittivity, *ω*_p_ is the plasmon resonance frequency of Al, and *γ*_p_ is the damping constant of Al in the Drude model. *s*_*j*_, *ω*_*j*_, and *γ*_*j*_ are the oscillation strength, resonance frequency, and damping constant for the Lorentz model, respectively. The damping constant *γ*_p_ can be written as *ρne*^2^/*m*_e_, where *ρ, n*, and *m*_*e*_ refer to the electrical resistivity, electron density, and effective electron mass. The electrical resistivity can be affected by intrinsic ohmic loss and extrinsic electrical scattering loss. In order to consider the scattering loss induced by different surface roughness, we divide the resistivity (*ρ*) into two terms; one is bulk resistivity (*ρ*_b_) and the other is surface resistivity (*ρ*_s_). Both parts of the resistivities are relevant to the grain boundary and surface roughness, which can significantly influence the damping constant in the Drude model. The bulk resistivities *ρ*_b_ of sample A and sample B are 0.22 and 2.65 *μΩ*cm, which are taken from references^16^,^17^. *ρ*_s_ is shown as^14^


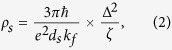


where *ħ* is the reduced Planck constant, *d*_*s*_ is the skin depth of the Al film, *k*_f_ is the Fermi wave vector, ∆ is the RMS height, and *ζ* is the lateral correlation length. Here we use the skin depth *d*_*s*_ of 8 nm to describe the decay thickness of the propagating electromagnetic wave that can influence the surface resistivity at a wavelength of 350 nm^1^. To get dielectric functions for Al, we calculate the value of the lateral correlation length *ζ* by fast Fourier transform (FFT) from the AFM image analysis software, NanoScope Analysis 1.5. The FFT spectra maps of sample A and sample B are shown in Fig. 1e,f, which are directly transformed from Fig. 1c,d. Since the lateral correlation length, ζ, is associated with the correlation function G(x, y), which is used for the description of the horizontal surface roughness, the roughness spectrum of the correlation function is given by the Fourier transform F[G(x, y)], which allows us to obtain the roughness parameters^11^ from the AFM data. We can then extract the lateral correlation lengths, *ζ*, for sample A and sample B to be 500 nm and 58.8 nm, respectively. The RMS heights, ∆, defined as the vertical surface roughness, for sample A and sample B are 0.653 nm and 2.5 nm. With other parameters in the Drude model taken from the references^18^,^19^,^20^, we can estimate the metal ohmic loss.

With eq. (2) the surface resistivities *ρ*_s_ of sample A and sample B can be calculated as 0.02 and 2.81 *μΩ*cm. It can be seen that the increase of *ρ*_s_ and *ρ*_b_, which arises from the electron scattering from the rough surface and grain boundary, results in the enhancement of the damping constant. In this way, *γ*_p_ of sample B is 22 times larger than sample A. Then, we use a numerical least square method to optimise the fitting curves of sample A and sample B. The fitting results are shown as solid lines in Fig. 1b, which fit extremely well to both measured sample A and sample B reflectivity spectra (See Table S1 in Supporting Information for the fitting parameters).

The fitting results show that the Lorentz terms exist in the visible and near infrared region, corresponding to the interband transitions of Al. On the other hand, *ε*_∞_, referring to the background polarisation of the material, varies in different samples^21^,^22^. In our fitting results, the larger *ε*_∞_ of sample B is due to lattice disorder in low quality processing, which can have an influence on the background polarisation of metallic samples. At short wavelengths, especially in the UV regime, the optical properties of metallic samples are sensitive to surface roughness, grain boundary features, and lattice quality, leading to a remarkable decline in the reflectivity of sample B. Nevertheless, further relations between the polarisation and surface roughness of materials should be explored in the future.

**Figure 2:**
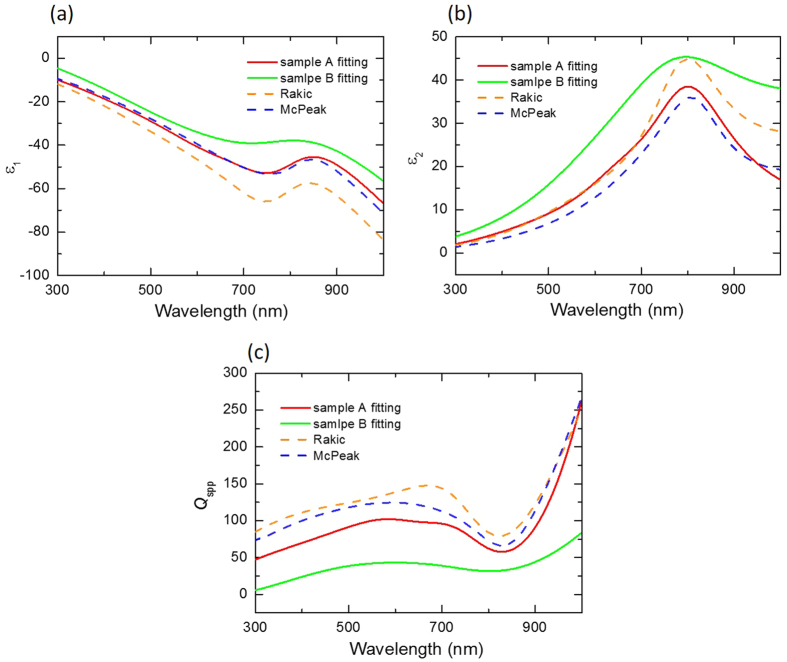
(**a**,**b**) are the real and imaginary parts of the permittivity of Al for our data and the data of the two references, respectively. The quality factor of the SPP mode is shown in (**c**).

The broadband permittivity of Al from our data and other references is shown in Fig. 2, where the two dashed lines represent the two sets of data studied by Rakic *et al*.^23^ and McPeak *et al*.^24^, respectively, and the two solid lines represent the data for sample A and sample B, respectively, calculated by the fitted parameters. The real and imaginary parts of the permittivity of Al are shown in Fig. 2a,b, respectively. In Fig. 2c, we introduce the quality factor for an SPP system, which is denoted as *Q*_SPP_, to evaluate the strength of local-field enhancement on the Al surface. The permittivity consists of the real and imaginary parts that represent the field distribution and loss, respectively. *Q*_SPP_ takes into account the contributions of both the real and imaginary parts of the permittivity in order to evaluate the quality of overall Al and is defined as^25^


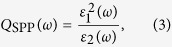


where *ε*_1_ and *ε*_2_ are the real and imaginary parts of the permittivity, respectively. We can quantify the quality of our sample A and sample B via the calculated values of *Q*_SPP_ to understand how the surface roughness influences SPP modes.

In Fig. 2a,b, we find that our data for sample A agree quite well with the data of the two references. Furthermore, compared with sample A, sample B, with higher surface roughness leading to larger *γ*_p_, has increased *ε*_1_ and *ε*_2_ for all the wavelengths we calculated. The *ε*_1_ and *ε*_2_ of sample B at the target wavelength of 372 nm are, respectively, 4.81 and 2.75 larger than the sample A. From Fig. 2c, we note that our data for calculation on sample A is also near the data of the two references, while our data for sample B is lower, in contrast with that of sample A due to the higher surface roughness. When the surface roughness of the Al film increases, the SPP mode propagating along the Al surface will be changed to the one that has larger intrinsic ohmic loss. The *Q*_SPP_ for sample A at the wavelength of 372 nm is 63.8, which is higher than the one for sample B (*Q*_SPP_ = 18.6). As a result, decreasing the surface roughness of Al films is a proper method to realise high-efficiency plasmonic devices.

### Characteristics of nanolasers

**Figure 3:**
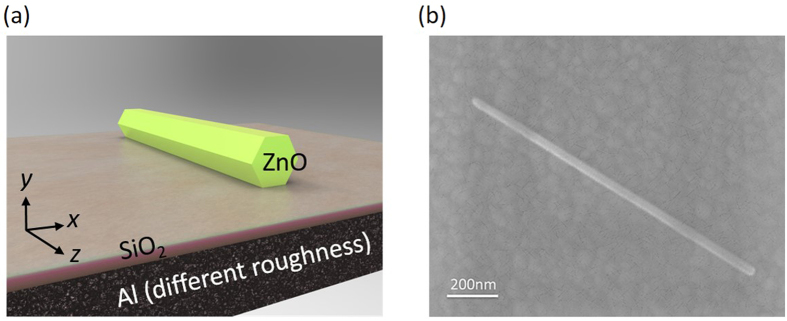
(**a**) The schematic diagram of an SPP nanolaser containing a ZnO nanowire on a SiO_2_-Al slab. (**b**) The SEM picture of a single ZnO nanowire lying on sample B film with a SiO_2_ spacer layer. The length of the ZnO nanowires is about 1.2 μm, and the growing direction lies along the c-axis of the wurtzite structure.

The dramatic drop in reflectivity at UV wavelengths on the roughened Al interface could lead to degraded SPP nanolaser performance. To investigate the lasing property at UV wavelengths, we use the ZnO nanowire as a gain medium with an emission wavelength in the 370−380 nm range to construct the SPP nanolaser. As a consequence, the UV ZnO SPP nanolaser is based on the metal-insulator-semiconductor (MIS) structure, consisting of a single-crystalline ZnO nanowire lying on a metallic Al film separated by a SiO_2_ dielectric spacer layer to greatly diminish the metal loss, as shown in Fig. 3a. The dielectric spacer layer not only controls the optical confinement, but can also isolate ZnO excitons from quenching at the metal surface. The metallic Al films can facilitate the generation of surface plasmons (SPs) and significantly affect the performance of plasmonic nanolasers. In this study, we deposit the ZnO nanowires on Al metal made from sample A and sample B. Figure 3b shows the SEM picture of ZnO nanowire lying on the Al film with a SiO_2_ spacer layer. The length of the ZnO nanowires was approximately 1 μm.

The permittivities of sample A and sample B calculated from the Drude-Lorentz model are used to analyse the performance of the UV plasmonic nanolasers. The calculated parameters that are relevant to lasing are listed in Table 1. Modal loss, waveguide confinement factor, and transparency gain of the guided plasmonic modes are calculated with 2D models while mode volume and threshold gain are calculated with 3D models. Their values are different for the sample A and sample B films in response to the contributions of different surface roughness.

The side length of the ZnO hexagonal cross section is about 30 nm (See Supporting Information Figure S2), and the diameter of the ZnO nanowire is smaller than the diffraction limit of dielectric guided mode. Because of this, there are no photonic modes inside it, and thus only SPP modes are induced. Furthermore, the ZnO nanowire is tiny enough that only one SPP mode (the fundamental mode) exists in the cavity since the contact area between the ZnO nanowire and SiO_2_ layer is too narrow along the *x* direction to confine higher order SPP modes. The apparent difference between the modal characteristics of SPP fundamental modes based on sample A and sample B in Table 1 is that the modal loss caused by sample B is about 2.7 times as much as the one caused by sample A, and then, the major cause described above leads to the fact that the transparency gain of a sample B-based cavity is 2.5 times larger than the one of a sample A-based cavity. This trend agrees with the result in Fig. 2c because sample B, with higher surface roughness, tends to encounter more extinction for SPP modes on the surface, which has been accounted for in the effective dielectric functions. On the other hand, the nanoscale roughness, which is smaller than the emission wavelength, results in only a minor difference between the mode profiles of sample A-based and sample B-based cavities. Therefore, the confinement factors of two different samples are almost the same. Including the mirror loss, the threshold gain of the sample B-based cavity is 1.6 times higher than that of the sample A-based cavity.

Furthermore, only a portion of the input optical energy is effectively coupled to the electromagnetic modal energy inside the active region of the cavity and consumed for overcoming the cavity losses. In order to estimate the pumping threshold *P*_th_, the pumping efficiency *η*_p_ is expressed as


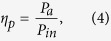


where *P*_in_ is the optical pumping power and *P*_a_ is the power absorbed in the gain medium. The ratio of threshold gains and pumping efficiencies *η*_p_ of the cavity modes is proportional to the corresponding pumping threshold *P*_th_ (See Supporting Information). The pumping efficiency of ZnO nanolasers on the sample A interfaces is 1.82 times higher than that on sample B interfaces at a pumping wavelength of 355 nm because of the influence of the effective permittivity function. This also indicates that a smooth metal surface will increase the efficiency of power absorption. To sum up all the factors, the pumping threshold *P*_th_ of ZnO nanolasers on sample A interfaces are calculated to be much lower (~33.1%) compared to those of the counterpart on sample B interfaces. Our theoretical analysis shows that metal-related losses owing to the roughness of the metallic interface have profound impacts on the performance of Al-plasmonic nanolasers in the UV wavelength regime.

**Figure 4:**
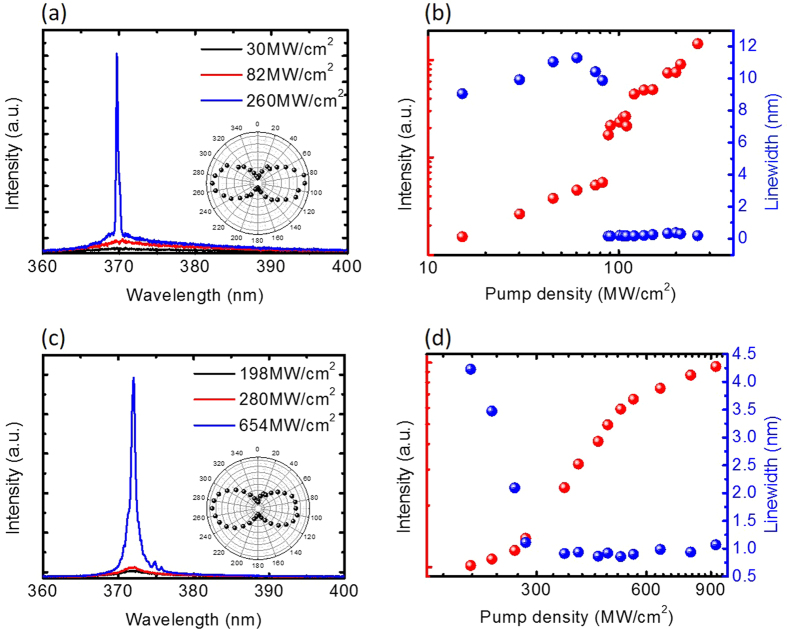
Lasing characteristics of an SPP nanolaser at 77 K. (**a**,**c**) The measurement spectrum of ZnO/SiO_2_/sample A and ZnO/SiO_2_/sample B. The inset shows a polarisation direction parallel to the nanowire. (**b**,**d**) The red sphere shows the corresponding light-light curve of (**a**,**c**), and the blue sphere shows the line width extracted from (**a**,**c**).

Figure 4 shows the optical performance measurements of ZnO devices with sample A and sample B films. Both of the samples show a wide spontaneous emission spectrum at low pumping power conditions. Sharp peaks were observed, with full-width-at-half-maximum of 0.5 nm, until the pumping density reached 85 MW/cm^2^ and 280 MW/cm^2^, as shown in Fig. 4a,c, respectively. The inset demonstrates that a polarisation direction of the emission is parallel to the nanowire after reaching the threshold condition, which indicates the longitudinal emission originated from the fundamental SPP mode^9^. The corresponding light-light curves are shown in Fig. 4b,d. The surface roughness and crystal quality of sample B induces high scattering loss and large intrinsic ohmic loss, and results in a threshold density about 3.3 times larger than the sample A device, which is consistent with the calculation results. (See Supporting Information Figure S3)

## Conclusion

We analysed the effects of surface roughness on UV ZnO SPP nanolasers fabricated on Al metal samples with different degrees of surface roughness. By using a modified Drude-Lorentz model in conjunction with surface roughness measurements, the dielectric functions of both sample A (grown by MBE) and sample B (grown by E-gun) can be fitted well to the measured reflectivity spectra. The surface roughness of sample A is 3.8 times smaller than that of Sample B, and its lateral correlation length is 8.5 times longer, which results in a 2.7 times smaller propagation length for the generated SPP wave. As a result, sample B with a higher surface roughness induces more electron scattering and light scattering for SPP modes, leading to a higher threshold gain of the plasmonic nanolaser. The experimental results agree pretty well with the calculations. We believe our study provides a simple and fast method to evaluate the characteristics of SPP waves on rough metallic interfaces and corresponding plasmonic nanolasers.

## Method

### Simulation

We investigated the influence of different surface roughnesses of Al metal via numerical simulation carried out by FEM using the COMSOL Multiphysics commercial package. The side length of the ZnO hexagonal cross section is 30 nm, and the thickness of the SiO_2_ dielectric layer is 5 nm. The thickness of the metal is 100 nm for the experiment, larger than the skin depth in our experimental wavelength range. Therefore, we use infinite thickness for the metallic substrate for our simulation. A perfectly matched layer (PML) surrounding the structural model is used to simulate the large substrate and space with respect to the size of the cavity. The simulation also incorporated the refractive indices of ZnO^26^ and SiO_2_^27^ along with their dispersive properties, and the refractive index of air is 1.

The waveguide confinement factor, Г_wg_, is expressed as the ratio of the modal gain to the material gain in the active region. It is defined as^28^,^29^


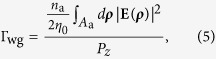


where *n*_a_ is the refractive index of the gain medium, *η*_0_ is the intrinsic impedance, *A*_a_ is the region of the gain medium, and *P*_*z*_ is the power flow in the propagation direction. The modal loss *α*_i_ can be determined by the imaginary part of the modal propagation constant *k*_*z*_ as *α*_i_ = 2 Im[*k*_*z*_]. Its inverse, *α*_i_^−1^, is denoted as the propagation length, *L*_p_, which describes how long the mode can propagate in a waveguide. The transparency gain, *g*_tr_, is defined as the gain of the mode that can propagate along a waveguide without attenuation and is expressed as *g*_tr_ = *α*_i_/Г_wg_. The mode volume, *V*_m_, which represents the special energy density of the cavity mode is expressed as^30^


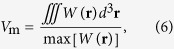


where *W*(**r**) is the local electromagnetic energy density at the position **r**. *W*(**r**) is defined as


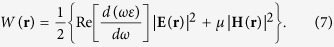


In addition to the modal characteristics (*α*_i_, Г_wg_, and *g*_tr_), calculations of the performance of Fabry-Perot cavities must consider the cavity length *L* and the reflectivity *R* of the mirrors at both ends of the cavity. Therefore, the threshold gain *g*_th_ of the cavity is defined as^28^,^29^


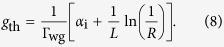


The pumping threshold *P*_th_ is defined as


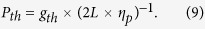


where *η*_p_ is the pumping efficiency. In order to estimate the pumping efficiency, we first use the transfer-matrix method to calculate the power flow through ZnO/SiO_2_/Al multilayered structures, where the permittivities of different Al metals are obtained from the fitted parameters in Fig. 2 (shown in the Supporting Information Figure S4). Then the ratio of pumping efficiency of sample A- and B-based structures is calculated by the power flow that is actually absorbed by the gain medium.

### Fabrication and pumping experiments

The sample A and sample B films were grown on a gallium arsenide (GaAs) (100) substrate by a molecular beam epitaxy system and an electron-gun evaporator, respectively. Because the construction of the sample A film strongly depended on the template morphology, we first grew a 200-nm-thick undoped GaAs buffer layer and let this serve as the ultra-flat template for the subsequent Al growth. In addition, before the substrate temperature cooled down to room temperature the surface was turned from an As-rich condition into a Ga-rich condition. During the cooling procedure, the wafer was kept in an ultrahigh vacuum chamber to prevent surface oxidation. While waiting for the residual arsenic pressure to be pumped down to less than 1 × 10^−10^ Torr, a 100-nm-thick Al layer was grown at ~0 °C with a growth rate of 0.05 nm/s.

After the metallic Al film preparation, we deposited a 5-nm-thick SiO_2_ dielectric spacer layer on the sample A and sample B films. Before the film deposition, the chamber pressure was about 3 × 10^−6 ^Torr and the growth rate was about 0.02 nm/s at 20 °C. The deposition rate and the film thickness were monitored in real time with a quartz crystal oscillator. Then we directly dispersed the ZnO nanowire powder on the patterned SiO_2_/sample A and SiO_2_/sample B substrates. Figure 3b shows the SEM picture of a single ZnO nanowire lying on the sample B film with a SiO_2_ spacer layer. The growing direction lies along the c-axis of the wurtzite structure. The detailed fabrication of ZnO nanolasers was reported previously^14^.

We placed both of the samples into a cryogenic chamber with ambient temperature controlled at T = 77 K. A Nd:YVO_4_ 355 nm pulse laser with 0.5 ns pulse width and 1 kHz repetition rate was launched into a near ultraviolet high magnification (100X, N.A = 0.55) objective lens and focused on the targeted sample with a small spot size, of diameter ~15 μm.

## Additional Information

**How to cite this article**: Chung, Y.-C. *et al*. Surface roughness effects on aluminium-based ultraviolet plasmonic nanolasers. *Sci. Rep.* **7**, 39813; doi: 10.1038/srep39813 (2017).

**Publisher's note:** Springer Nature remains neutral with regard to jurisdictional claims in published maps and institutional affiliations.

## Supplementary Material

Supporting Information

## Figures and Tables

**Table 1:** Parameters for the sample A-based and sample B-based cavities relevant to lasing.

	*α*_i_ (cm^−1^)	Γ_wg_	g_tr_ (cm^−1^)	*V*_m_ (λ^3^)	*g*_th_ (cm^−1^)
sample A	2.70 × 10^4^	0.387	6.98 × 10^4^	2.63 × 10^−2^	1.48 × 10^5^
sample B	7.19 × 10^4^	0.413	1.74 × 10^5^	2.53 × 10^−2^	2.47 × 10^5^
