# Supplementary material for: Surface roughness effects on aluminium-based ultraviolet plasmonic nanolasers
Source: Sci Rep. 2017 Jan 3;7:39813. doi: 10.1038/srep39813 (PMC5206644; doi:10.1038/srep39813)
Supplement: Supporting Information [file srep39813-s1.doc]

**Supporting Information**

**Surface roughness effects on aluminium-based ultraviolet plasmonic nanolasers**

Yi-Cheng Chung^1^, Pi-Ju Cheng^2^, Yu-Hsun Chou^3,4^, Bo-Tsun Chou^5^, Kuo-Bin Hong^4^, Jheng-Hong Shih^1^, Sheng-Di Lin^5^, Tien-Chang Lu^4,#^ & Tzy-Rong Lin^1,6,*^

^1^National Taiwan Ocean University, Department of Mechanical and Mechatronic Engineering, Keelung 202, Taiwan

^2^Academia Sinica, Research Center for Applied Sciences, Taipei 115, Taiwan

^3^National Chiao Tung University, Institute of Lighting and Energy Photonics, Tainan 711, Taiwan

^4^National Chiao Tung University, Department of Photonics, Hsinchu 300, Taiwan

^5^National Chiao Tung University, Department of Electronics Engineering, Hsinchu 300, Taiwan

^6^National Taiwan Ocean University, Institute of Optoelectronic Sciences, Keelung 202, Taiwan

Corresponding authors: ^*^trlin@ntou.edu.tw; ^#^timtclu@mail.nctu.edu.tw

**Table**

**Table S1.** Fitting parameters of the Drude-Lorentz model.

units of ω and γ: rad/s

|  | ε_inf_ | ω_p_ | γ_p_ | s_j_ | ω_j_ | γ_j_ |
| --- | --- | --- | --- | --- | --- | --- |
| sample A | 2.3 | 1.83×10^16^ | 1.12×10^13^ | 8.32 | 3.47×10^15^ | 4.07×10^15^ |
|  |  |  |  | 7.56 | 2.36×10^15^ | 6.43×10^15^ |
|  |  |  |  | 2.22 | 2.89×10^15^ | 9.87×10^15^ |
| sample B | 8.8 | 1.83×10^16^ | 2.53×10^14^ | 7.61 | 2.43×10^15^ | 1.02×10^15^ |
|  |  |  |  | 9.05 | 3.77×10^15^ | 8.15×10^15^ |
|  |  |  |  | 12.21 | 2.88×10^15^ | 2.25×10^15^ |

**Figure**

**Figure S1.** The reflectivity spectra of fixed artificial aluminium metals with different RMS roughness. To investigate the influence on interfaces of aluminium films with different kinds of roughness (sample A and B) due to Rayleigh scattering, the formula of reflectivity in Ref. [S1] is considered, which is affected only by Rayleigh scattering. For simplifying the analysis, the reflectivity depends only on the incident wavelength and the RMS height of the surface roughness. Here, the reflectivity of a totally smooth surface of an artificial metal is assumed to be 0.9 for wavelength ranged from 200 nm to 1000 nm. Different degree of roughness is then applied to show the effect of Rayleigh scattering. The result shows that only when the RMS value of the surface exceeds 5 nm, the Rayleigh scattering starts to play important role at short wavelength range. Therefore, in both of our samples, the relatively small RMS values of the surfaces shall not dominate in the reflectivity drop at the short wavelength band.

**Figure S2.** The average side length of different ZnO nanowires. To estimate the side length of a single nanowire, we measure the diameter of the nanowire on the SEM picture and calculate the side length by the geometric relation of the hexagon. We estimate the average side length of a single nanowire from three cross sections with similar diameters and without attached particles on the nanowire. Due to the error caused by the uncertainty of measurement, the error bar was added and was about 1.8 nm. We choose the side length of ZnO nanowire as 30 nm in our simulation process.

**Figure S3.** The threshold power of different nanolaser devices. Here, we measure 10 nanolaser devices based on ZnO/SiO_2_/sample A and ZnO/SiO_2_/sample B structures. The average threshold power of sample B-based nanolaser devices is 425 MW/cm^2^, and sample A-based nanolaser devices is 119 MW/cm^2^. Due to the large loss caused by the surface roughness effect in sample B interface, the average threshold power is about 3.6 times larger than that of the sample A-based nanolaser device. The statistic result is consistent with the result in our main manuscript.


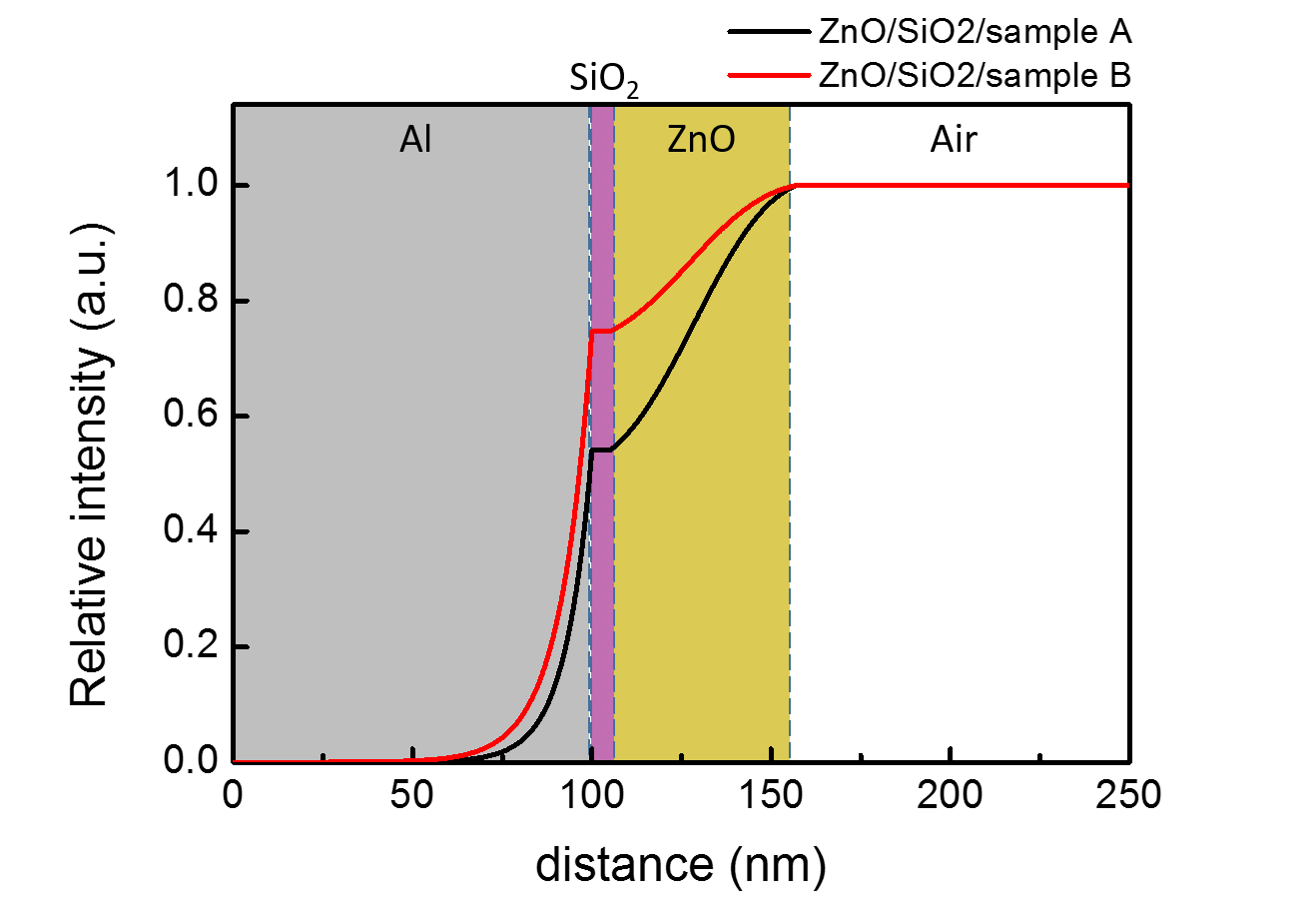


**Figure S4.** The distribution of power flow in ZnO/SiO_2_/Al multilayered structures. To estimate the pumping efficiencies of plasmonic nanolasers based on aluminium films with different roughness (sample A and B), electric magnetic plane waves are normally incident from air to ZnO/SiO_2_/Al multilayered structures. The thicknesses of ZnO, SiO_2_, Al layers are 52, 5, 100 nm. The solid curves represent normalized power flows declining with the distance to air/ZnO interfaces. The decrease of power flow toward the metal is due to the absorption of the layer. Therefore, the absorbed power in the ZnO nanowire can be estimated by the power difference between the input and exit plane. The ratio of pumping efficiency of sample A- and B-based structures are calculated by the descent of power flow in ZnO layer, which is actually absorbed by the gain medium.

**Reference**

1. Endriz, J. G. & Spicer, W. E. Study of aluminium films. I. Optical studies of reflectance drops and surface oscillations on controlled-roughness films. Phys. Rev. B 4, 4144 (1971).
